# Large-Scale Recurrent Neural Network Based Modelling of Gene Regulatory Network Using Cuckoo Search-Flower Pollination Algorithm

**DOI:** 10.1155/2016/5283937

**Published:** 2016-02-16

**Authors:** Sudip Mandal, Abhinandan Khan, Goutam Saha, Rajat K. Pal

**Affiliations:** ^1^Department of Electronics and Communication Engineering, Global Institute of Management and Technology, Krishna Nagar, West Bengal 741 102, India; ^2^Department of Computer Science and Engineering, University of Calcutta, Kolkata 700 098, India; ^3^Department of Information Technology, North-Eastern Hill University, Shillong 793 022, India

## Abstract

The accurate prediction of genetic networks using computational tools is one of the greatest challenges in the postgenomic era. Recurrent Neural Network is one of the most popular but simple approaches to model the network dynamics from time-series microarray data. To date, it has been successfully applied to computationally derive small-scale artificial and real-world genetic networks with high accuracy. However, they underperformed for large-scale genetic networks. Here, a new methodology has been proposed where a hybrid Cuckoo Search-Flower Pollination Algorithm has been implemented with Recurrent Neural Network. Cuckoo Search is used to search the best combination of regulators. Moreover, Flower Pollination Algorithm is applied to optimize the model parameters of the Recurrent Neural Network formalism. Initially, the proposed method is tested on a benchmark large-scale artificial network for both noiseless and noisy data. The results obtained show that the proposed methodology is capable of increasing the inference of correct regulations and decreasing false regulations to a high degree. Secondly, the proposed methodology has been validated against the real-world dataset of the DNA SOS repair network of* Escherichia coli*. However, the proposed method sacrifices computational time complexity in both cases due to the hybrid optimization process.

## 1. Introduction

A gene regulatory network (GRN) represents the regulatory behaviour or dependencies among a group of genes inside a cell. A GRN is characterized by a directed graph in which nodes denote genes, and regulatory dependencies among genes are depicted by directed edges between the corresponding nodes. There are two types of interaction, namely, activation and inhibition. This kind of network is unique for particular functions within a cell. Thus, the study of GRN is essential to ascertain the genetic causes of a particular disease. As a consequence of this, scientists can venture into the development of new and improved techniques for the treatment of a disease [1].

Nowadays, DNA microarrays [2, 3] are extensively utilized for the investigation of finding reasons for different illnesses. A microarray dataset contains the gene expression levels of millions of genes of a species under investigation, at a particular condition or set of conditions. Time series microarray database consists of changes in the expression of genes with time in response to some disease-causing events or any form of treatment at different time points. This includes essential information regarding the dynamic behaviour as well as the dependencies of genes.

In this work, Recurrent Neural Network (RNN) [4] which is a closed loop Neural Network with a delayed feedback has been used to model dynamics and dependencies of the genetic system from temporal genetic data. Using suitable optimization methods, RNN based GRN can be inferred for the purpose, where the objective function of optimization is chosen so that it becomes proportional to the training error. Different metaheuristics techniques, namely, Genetic Algorithm (GA) [5], Particle Swarm Optimization (PSO) [6], *K*-Means Population-Based Incremental Learning (KPBIL) [7], Invasive Weed and Artificial Bee Colony (ABC) [8], PSO and Ant Colony Optimization (ACO) [9], Bat Algorithm [10], have been implemented to infer GRNs from the time series microarray data. Computational reconstruction of GRN is essentially a reverse engineering problem. All the approaches mentioned above are tested against both small-scale artificial and real-life GRNs. However, Noman et al. [11] proposed Decoupled Recurrent Neural Network, which was trained by Differential Evolution (DE), where a penalty term or L1 regularizer was introduced into the objective function to balance between the accuracy of the parameters and the actual network structure. The model mentioned above [11] is very efficient in finding all the valid regulations and accurately reproducing the dynamics of both of the small networks studied. However, the main disadvantage of this model is that the given model predicts a large number of false regulations for more extensive or larger networks. The balance between fitness value and actual network structure for large-scale networks is the primary concern of the reverse engineering problem of GRN, and it is still an open area of research.

In this paper, a hybrid Cuckoo Search (CS)-Flower Pollination Algorithm (FPA), CS-FPA, is proposed for the inference of GRNs from time-series data. FPA is used to train the RNN parameters, and CS is introduced to find the biologically plausible network architecture to select the best combination of genes that are responsible for modifying the expression of each gene. The preliminary notions of RNN, CS, and FPA are discussed in the next section. The details of the fitness function of FPA for decoupled RNN and the learning process for finding the actual structure of a GRN using CS are discussed in Section 3. Next, the effectiveness of the proposed CS-FPA based RNN model is tested against a large artificial GRN without the presence of noise as well as with noisy data. The result is also compared with other state-of-the-art methods. The conclusion is given in Section 4.

## 2. Theoretical Background

We have discussed the fundamental theoretical concepts of RNN, CS, and FPA, in this section, for a better understanding of the proposed methodology.

### 2.1. Preliminaries of RNN

**Figure 1:**
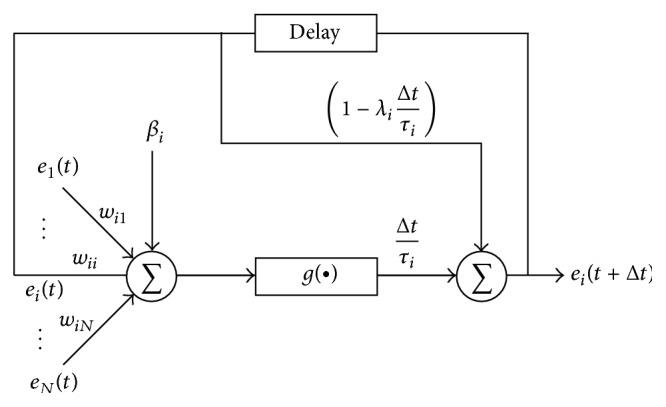
A neuron in the RNN model [7].

The RNN model [12] is a closed loop Artificial Neural Network that has a delay variable, between the outputs of each neuron in the output layer of the RNN, to each of the neurons in the input layer, which is suitable to model temporal behaviour or dynamics of data (Figure 1). For a canonical RNN model [4–9, 11] it is assumed that each of the total *N* output neurons in the unit is a gene expression value of next time instant *e*_*i*_(*t* + Δ*t*), and the input neurons are the gene expression of present state *e*_*i*_(*t*) for the same genes; thus they interact with each and every one in regenerative way:

(1) eit+Δt=Δtτif∑j=1Nwi,jejt+βi+1−Δtτieitwhere  i=1,3,…,N.

Here, *f*(·) is usually a sigmoid function; *f*(*z*) = 1/(1 + *e* ^−*z*^) is used as a classification function; and *w*_*i*,*j*_ is the weight of inputs of the RNN model, and it stands for the type and strength of the regulatory interaction of the *j*th gene with the *i*th gene. From the point of view of a GRN, each node corresponds to a gene, and a connection between two nodes defines their interaction. The weight values can be either positive, negative, or zero; *w*_*i*,*j*_ is the most significant term for a GRN as the value of *w*_*i*,*j*_ is the connecting weight of an edge of the GRN, which represent the connections between gene-*i* and gene-*j*. A positive value of *w*_*i*,*j*_ represents activation of gene-*i* by gene-*j*, a negative value denotes repression or inhibition of gene-*i* by gene-*j*, and *w*_*i*,*j*_ = 0 means that gene-*j* has no regulatory control on gene-*i*. The term *β*_*i*_ represents the basal expression level or a bias term, and *τ*_*i*_ is a time constant (delay) of the *i*th gene; Δ*t* is incremental time instance; in this work it is always set as 1. Thus, any RNN model can be expressed by a set of *N*(*N* + 2) parameters, *Ω* = {*w*_*i*,*j*_, *β*_*i*_, *τ*_*i*_}, where *i*, *j* = 1,2,…, *N*.

### 2.2. Preliminaries of Cuckoo Search (CS) Optimization

Yang [13] first proposed Cuckoo Search optimization [14–18] based on brood parasitism of cuckoo birds that reproduce their eggs by utilizing nests of other host birds. These birds have the ability to use other birds for raising the new generation. Cuckoo lay their eggs, one or more than one, in the nest of the host birds in their absence using Lévy flight. Lévy flight [19] is an important characteristic of CS. Levy flight is defined as a random movement done by the birds with a step value of distributed probability. While new solution *x* ^*t*+1^ for a cuckoo *i*, Lévy flight is performed as

(2) $$\begin{array}{c} x_{i}^{t+1}=x_{i}^{t}+\alpha ⊕L\overset{´}{e}vy\left(\;\lambda \;\right). \end{array}$$

Here *α* > 0 is the step size; ⊕ means entrywise multiplications. Levy flights essentially provide a random walk and the random steps are drawn from a Lévy distribution [13, 19] as follows:

(3) $$\begin{array}{c} L\overset{´}{e}vy\left(\;\lambda \;\right)\sim \frac{\lambda \Gamma \left(\lambda \right)\sin\left(\pi \lambda /2\right)}{\pi }\frac{1}{s^{1+\lambda }}. \end{array}$$

In this equation, Γ(*λ*) is the standard gamma function. This distribution is valid for large steps *s* > 0. For optimization problems, one cuckoo nest corresponds to one solution of an optimization problem. When the host bird recognizes the alien eggs, the host bird may destroy the eggs or may leave its nest and build a new nest with a certain probability *p*_*a*_. To avoid this, cuckoos learn to make eggs similar to the host bird's eggs. However, the highest quality nest with eggs (i.e., best solutions) will be selected to move over to the next generation where the quality of an egg or fitness of a solution is simply proportional to the value of objective function.

### 2.3. Preliminaries of Flower Pollination Algorithm (FPA)

FPA [20] is typically associated with the transfer of pollen for reproduction or flowering of plants, and pollinators such as insects, birds, and bats are mainly responsible for such transfer. FPA [21–23] is a recently proposed metaheuristic that is based on some simplified rules for pollination. Biotic cross-pollination can be assumed as a process of global pollination, and pollen carrying pollinators follow Lévy flights during transport (Rule  1). For local pollination, abiotic pollination and self-pollination are used (Rule  2). Pollinators may develop flower reliability, which is proportional to the resemblance of two flowers, that is, reproduction probability (Rule  3). The switching of local to global pollination can be controlled by a switch probability *p* ∈ [0,1], slightly biased towards local pollination (Rule  4). Here, each pollen or flower corresponds to a solution of the optimization problem being considered.

Global and local pollination (i.e., search) are done according to the following two equations [21], respectively:

(4) xit+1=xit+γ Le´vyλg∗−xit,

(5) xit+1=xit+εxjt−xkt.

Here, *x*_*i*_^*t*^ is the pollen *i* or solution vector *x*_*i*_ at iteration *t*, *γ* is the scaling factor to control the step, *g*_*∗*_ is the current best solution found among all solutions at the current iteration, *x*_*j*_^*t*^ and *x*_*k*_^*t*^ are pollens from the different flowers of the same plant species, and *ε* stands for random walk step size within a uniform distribution in [0,1]. The reason behind selecting FPA as optimization method is that it gives better convergence and accuracy than other popular metaheuristic techniques [21].

## 3. Methodology

The RNN formalism is based on a set of parameters, *w*_*i*,*j*_, *β*_*i*_, *τ*_*i*_, which we refer to as RNN model parameters in this work. The reverse engineering of GRNs from temporal microarray data requires finding the optimum values of the RNN parameters with the help of optimization techniques such that the training error is minimized.

### 3.1. Model Used for Validation

We choose a large artificial network with 30 genes, to investigate the inference capability of the proposed algorithm. This network structure is very sparse in nature, and it has already been studied in [11]. The parameters of this architecture were chosen arbitrarily as shown in Table 1 where there are only 36 connections or regulations in the network; for example, *w*_1,14_ = −15, signifying an edge (a regulatory relationship) that exists between Gene 1 and Gene 14 in the GRN, and the negative value implies that Gene 14 inhibits or suppresses the expression of Gene 1. Any negative value denotes inhibition or repression while a positive value implies activation. The numerical value has only mathematical significance for the RNN formalism implemented here. Only the nature of the value of *w*_*i*,*j*_, that is, whether *w*_*i*,*j*_ < 0, 0, or >0, has biological significance.

The parameters *β*_*i*_ and *τ*_*i*_ also have no significance in the biological structure of a GRN. They are required for the mathematical modelling of the RNN formalism. They basically determine the bias and rate of change of dynamics for a particular gene. Using (1), 5 time series datasets, with 50 time points in each, are generated for learning of RNN assuming that Δ*t* = 1. These time series data are used as the training data, represented by a gene expression matrix where each column indicates a gene and each row indicates a time point; the data in each cell means the gene expression level of a particular gene at a particular time point.

**Figure 2:**
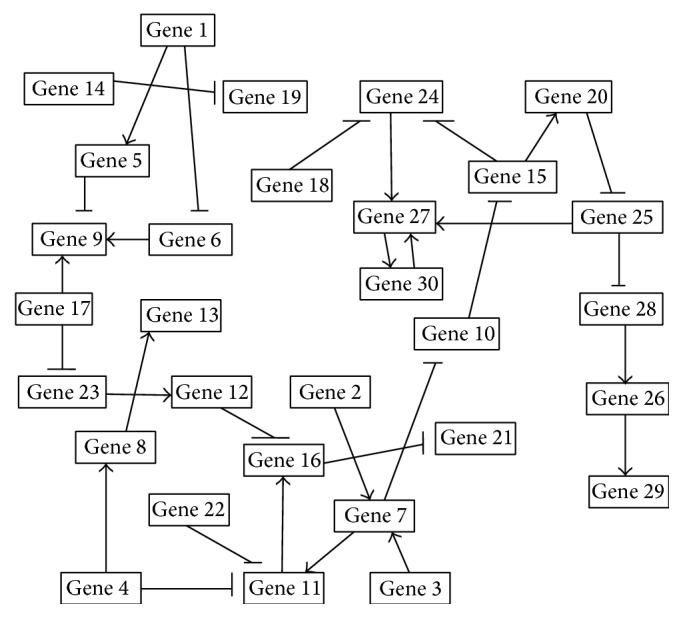
GRN using RNN parameters as described in Table 1.

The corresponding GRN is shown in Figure 2, where arrow-head and T-head denote activation and suppression, respectively, for positive and negative weights, respectively, between any two genes.

### 3.2. Decoupled Recurrent Neural Network Training

In all optimization methods, an objective/fitness function is used to measure the quality of a solution. One of the most regularly implemented estimation criteria is the squared error that is defined as follows:

(6) $$\begin{array}{c} fob=\overset{M}{\underset{k=1}{\sum }}\;\overset{N}{\underset{i=1}{\sum }}\;\overset{T}{\underset{t=1}{\sum }}{\left(\;e_{cal,k,i,t}-e_{exp,k,i,t}\;\right)}^{2}. \end{array}$$

In the above equation, *N* is the number of genes in the GRN, *T* is the number of sampling instances of the observed gene expression data, and *M* is the number of training datasets; *e*_cal,*k*,*i*,*t*_ is the numerically calculated gene expression value of the *i*th gene at time *t*, for the *k*th dataset; *e*_exp,*k*,*i*,*t*_ is the actual gene expression level of the *i*th gene at time *t*, for the *k*th dataset. The function *fob* represents the total squared error between the calculated and experimental gene expression data.

Thus, the training of the RNN model parameters is, in essence, a nonlinear optimization problem. The primary objective is to determine the optimal RNN parameters such that the mean square error is minimised, and the calculated gene expression data fits with the observed gene expression data. Since, for *N* genes, *N*(*N* + 2) parameters must be determined to find the solution of a set of equations as in (1), the RNN model parameter search space is of *N*(*N* + 2)-dimensional space (here 960). However, this space becomes too computationally expensive in the case of large-scale genetic networks. Therefore, the problem of inferring a GRN from temporal expression data is decoupled or divided into several subproblems corresponding to each of the genes. Now, the objective of each subproblem corresponding to *i*th gene is to find the values of decoupled RNN model parameters which minimizes the prediction. Thus, we define the objective function for the *i*th gene only as

(7) $$\begin{array}{c} {fob}_{i}=\overset{M}{\underset{k=1}{\sum }}\;\overset{T}{\underset{t=1}{\sum }}{\left(\;e_{cal,k,i,t}-e_{exp,k,i,t}\;\right)}^{2}. \end{array}$$

Hence, to solve the differential equation (1), the number of RNN parameters needed to determine is only (*N* + 2) parameters for the *i*th gene only. Thus, this decoupling method divides a *N*(*N* + 2)-dimensional problem *N* subproblems of dimension (*N* + 2). Accumulating the (*N* + 2) parameters of all *N* genes, the overall structure of the RNN model can be achieved which in turn will define the inferred GRN.

### 3.3. CS and FPA Hybridization

Real-life GRNs are sparsely connected; that is, very few connections exist among genes, and the measured data are also very noisy. Thus, for GRN inference problems, it is expected that the values of a majority of the weight parameters are zero. Nevertheless, it is found that though the complexity is reduced up to a certain limit by using a decoupled strategy, it may lead to different solutions. Each solution may have very low error value depending on the different connectivities or structures among the genes in the GRN and the corresponding values of the kinetic parameters. This is known as the overfitting problem that occurs mainly due to the nonlinearity of the RNN methodology, large search space, and a large number of parameters (here 32 for the decoupled RNN formalism, i.e., still quite large) to be optimized. Thus, to overcome this problem in real-life genetic networks, a balance between prediction error minimization and actual regulatory structure of GRN needs to be achieved.

The proposed Cuckoo Search-Flower Pollination Algorithm hybrid approach utilizes a single-layer RNN formalism to determine the weights of the edges between the input and output layers of RNN, with the help of FPA that minimizes the error between the calculated and experimental gene expression profiles. This RNN structure is hybridized with a new metaheuristic CS algorithm for creating hypothetical interconnections or regulatory edges among genes by selecting the best combination of affecting/regulatory genes. Moreover, the maximum connectivity of each gene is restricted to *I*_max_ as it is observed that real-life GRNs are sparsely connected; that is, very few genes participate in actual regulations.

In our present problem, we have chosen the value of *I*_max_ = 3; that is, we assume that at most any three genes can affect any single target gene. CS technique has been introduced to detect these three regulatory genes, and FPA is used to know the weight of the RNN model where these three regulatory genes act as the input node of RNN and the target gene acts as the output node. The RNN is implemented to determine how the gene expression levels of those three genes, at a particular time point, affect the target gene's expression value at the next time instance. Assuming this, we assign the value of *w*_*i*,*j*_ to be zero except for those three regulatory genes and the search space thus becomes 5-dimensional (3 weight, 1 bias, and 1 delay parameter). It may be possible that, for a particular regulatory gene set, one or more *w*_*i*,*j*_ becomes zero after optimization using FPA. This implies no regulation for those genes with zero value of *w*_*i*,*j*_.

The CS is initialized randomly with a population of different solutions or eggs on the different random nest, and the quality of each egg in the host nest is calculated using FPA based RNN to observe the impact of regulatory genes on other genes in the GRN as part of one generation. However, we initialized with a population of 10, each consisting of 3 different combinations of genes starting from (1, 2, 3), (4, 5, 6),…, (28, 29, 30). This type of predefined initialization is used as it increases the probability of covering all regulatory genes without repetition. Search range for the model is chosen as previous work [11] like *w*_*i*,*j*_ = [−25,25], *β*_*i*_ = [−10,10], and *τ*_*i*_ = [0,15], respectively.

The quality of a host nest or fitness of a solution is simply proportional to the objective function that is the resultant error of RNN for the set of particular genes or nest. FPA always tries to minimize the training error by optimizing the value of the RNN model parameters. During training of RNN, each pair in the training dataset contains the gene expression values for only 3 regulatory genes from the one time instance of the microarray data to be the input values to the RNN model, and the expression value of the target gene at the next time instance of the microarray data is the target output of the RNN. This helps to reduce the execution time by reducing the unnecessary calculations corresponding to nonregulatory genes. Moreover, it is observed that during optimization if the fitness value for FPA based RNN becomes less than 1 × 10^−8^; then it gives the almost actual value of the parameters of RNN for the regulatory genes. Therefore, a stopping criterion is introduced to minimize the execution time of the algorithm; that is, if the fitness value for a particular gene set becomes less than 1 × 10^−8^ after some iterations, the program stops execution instantly. Moreover, the corresponding genes set becomes the desired output.

Sometimes, it is found that if any solution does not consist of the actual regulatory genes, then the convergence is very slow which may increase the execution time as it is not able to go below the value 1 × 10^−8^ even after a large number of iterations. Therefore, another alternative stopping criterion is also imposed which can be stated as follows: if the difference between current best fitness value and fitness value of the (current-200)th iteration is less than 1 × 10^−10^, then the program execution will also stop. Better host nest with better quality eggs of each generation will move to the next generations. After successful completion of all iterations, we have a set of regulatory genes which can affect the target gene most. This process is repeated for all 30 genes one by one to get the final GRN structure.

Here inputs are the time series data, generated by (1), using parameters as shown in Table 1, and the outputs are a combination of 3 regulatory genes (*j*th) for a particular target gene (*i*th) along with the value of regulatory weights, that is, *w*_*i*,*j*_, corresponding to those 3 regulatory genes for which the minimum or optimum fitness value is achieved. It is worth mentioning that for a target gene we always get a combination of three responsible genes, but this does not mean that during GRN reconstruction there will always be regulatory edges from those regulatory genes towards target gene. The existence of a directed edge in a network also depends on the weights between target and regulatory genes. If amplitudes of weights are zero or very small, there will be no edges for the target gene; that is, there will be noninteraction. Small perturbation of obtained weights from actual one can be ignored unless and until it does not change the polarity or sign, that is, the type of regulation. Moreover, small values of weights can be considered as zero. It is believed that if the number of iterations and population size are large enough, these small perturbations can also be avoided, but execution time will also increase consequently. Thus, during reconstruction of GRN, both the set of regulatory genes and the value of the corresponding gene's weights must be kept in mind. The pseudocode of this CS-FPA hybrid is given as in Algorithm 1.

### 3.4. Results for Artificial Dataset

After execution of the proposed methodology, we get the hypothetical interconnections among genes regarding weights of the RNN model. Now, the performance of any inference algorithm for GRN is measured regarding sensitivity (*S*_*n*_), specificity (*S*_*p*_), accuracy, and Matthews Correlation Coefficient (MCC) [11, 24] which are defined as follows:

(8) Sn=TPTP+FN,Sp=TNTN+FP,Accuracy=TP+TNTP+TN+FP+FN,MCC=TP×TN−FP×FNTP+FP×TP+FN×TN+FP×TN+FN.

TP (True Positive) denotes the number of correctly predicted regulations, and TN (True Negative) represents the number of properly predicted nonregulations. FP (False Positive) denotes the number of incorrectly predicted regulations, and FN (False Negative) represents the number of falsely predicted nonregulations by the inference algorithm. All the experiments have been performed using MATLAB R2009b, running on Windows 7 with an Intel© Dual Core processor and 2 GB of RAM. In this work, both noiseless and noisy data are considered for the construction of the artificial network to validate the proposed algorithm. Validation of the proposed method is achieved in terms of correct prediction of RNN model parameters, that is, correct prediction of regulations in the GRN. The artificial time series datasets generated using (1) are used as training inputs.

For the reconstruction of a GRN consisting of 30 genes, we need an adjacency matrix (here weight matrix of RNN) of 30 × 30 dimension. In this case, there are only 36 regulations, that is, 36 nonzero value of weights (see the Supplementary Material available online at http://dx.doi.org/10.1155/2016/5283937). For noiseless data, using RNN model, we found that the proposed algorithm can predict 32 regulations correctly compared to those which were present in actual GRN, and also includes only four unwanted regulations. Thus, TP = 32, FP = 4, FN = 4, and TN = 860. It is found that the proposed algorithm is able to detect maximum correct regulations or TPs with a very good accuracy (for maximum cases, the fitness value went below 10^−8^). Though this method only detects 4 false regulations, 4 true regulations are also missing. Nevertheless its inference capability or performance is quite satisfactory and better than other state-of-the-art methods like DE [11].

Next, we add 5% random noise to the initial training dataset and apply the CS-FPA hybrid algorithm to it to check the robustness against noise like real-world data. It is observed that, in the presence of noise, the number of FPs increased significantly. However, it can still identify all the TPs (previously inferred) with good accuracy. For noisy data, TP = 32, FP = 19, FN = 4, and TN = 849.  Table 2 shows a comparative study of the performance of the proposed methodology with earlier work with respect to specificity and sensitivity.

It is quite interesting to observe that performance (in terms of sensitivity) of CS-FPA does not change for detecting true positive regulations in the presence of noise whereas sensitivity of DE [11] drastically falls to very low value in the presence on noise that denotes inability of DE [11] for finding actual regulation in the presence of noise. Moreover, specificity, accuracy, and MCC are better than DE [11], and values of these parameters do not change significantly due to noise though some false regulations are included into the GRN. However, due to a parallel implementation of two metaheuristic for one problem, overall computational time complexity is still significant although several break conditions are applied to minimize the execution time as much as possible which is 1.5 hours on average for this large network model.

### 3.5. Results for Real-Time Dataset of* E. coli*

**Figure 3:**
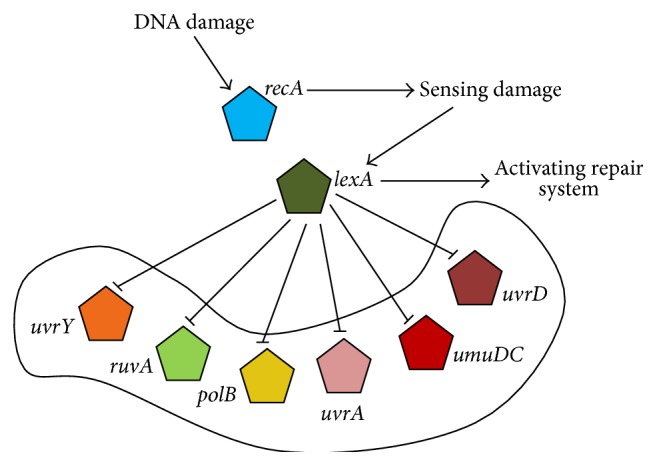
The graphical representation of the actual SOS network for* E. coli* [7].

Microarray experiments on the SOS DNA repair network for* E. coli *[25] were first done by the Uri Alon group [26]. The experimental datasets are considered as the benchmark for the evaluation of algorithms for reverse engineering of GRNs from real-world datasets. In the SOS network, 8 genes were considered (*uvrD, lexA, umuD, recA, uvrA, uvrY, ruvA, *and* polB* as shown in Figure 3) due to their significant involvement in the process of DNA repair. During their experiments, the* E. coli* cells were irradiated with UV light. Four experiments were performed with different UV light intensities. Each experiment consists of 50 time steps spaced by 6 minutes for each of the eight genes. However, in this work, the first dataset with all eight genes has been considered where it is being preprocessed by neglecting first time point (zero) normalizing in the range [0,1].

Search range for the model is chosen as previous work [11] like *w*_*i*,*j*_ = [−10,10], *β*_*i*_ = [−10,10], and *τ*_*i*_ = [0,10], respectively. For CS, the value of *N* is set as 2 as the number of available genes is limited to 8 only and other parameter settings remain the same. If we apply CS-FPA hybrid to the SOS dataset, only 4 out of 9 potential regulations can be appropriately predicted from the data, which are the inhibition of* lexA* on* uvrD*,* umuD, recA, *and* ruvA*. Moreover, it also includes 11 false regulations in the network which is a disadvantage of this process. The results are shown in Table 3.

Furthermore, using two or more time series does not yield any enhancement for the results. The cause behind this is that inference of real-time GRNs is an ill-posed problem that has no unique solution. Noise and measurement error in this type of real-time series microarray is another issue. This inherent difficulty is a limitation of our proposed method.

## 4. Conclusion

Various researchers have already proposed numerous techniques to solve the reverse engineering problem of GRNs from temporal genetic expression data in the domain of computational biology and bioinformatics. It is imperative to enhance the accuracy of the inference algorithms as well as to reduce the number of incorrect predictions (i.e., FPs) within a plausible runtime.

The RNN formalism is a very popular candidate for inferring GRNs from microarray gene expression data regarding biological plausibility and computational efficiency. In this work, we have implemented the decoupled RNN model where the regulatory parameters of each gene are calculated independently in separate search instances. We incorporated hybridized technique where two metaheuristics are paired to obtain the RNN based GRN model with less search space, less computational complexity, and more accuracy. In this paper, hybridized CS-FPA is proposed for reconstruction of GRNs where FPA is used to train the RNN parameters, and CS is introduced to select the best combination of genes that are responsible for modifying the expression of each gene. Moreover, the maximum connectivity of each gene is restricted to *I*_max_ as it is observed that real-life GRNs are sparsely connected; that is, very few genes participate in regulations.

To prove the efficiency of this inference algorithm, it is applied to a benchmark problem of the artificial network with 30 genes with and without noise. With the use of fewer data points, CS-FPA based RNN can infer the network with very high accuracy. However, in the presence of noise, the number of FPs increases significantly, but it can still identify all TPs (inferred in the noiseless scenario) with good accuracy. It is also found that noise robustness is better than other existing methods for artificial data. In the instance of the* E. coli* dataset, it can detect only four true regulations and includes some false regulations.

Another important observation that is apparent from our results is that the proposed methodology can reconstruct the large artificial GRNs more efficiently than that of real-life GRNs. However, this needs further study on different networks available to us, and the existing boundary of our work validates this observation. In the future, various regularization techniques, the inclusion of prior knowledge about GRNs, and parallel computing methods may be utilized to improve the accuracy and speed further.

## Supplementary Material

We have included the following in the supplementary materials:
(i) The noiseless dataset of the artificial network, used for training, and the corresponding results.(ii) The dataset of the artificial network with 5% noise added, and the corresponding results.(iii) The dataset of the *E. coli* DNA SOS repair network, and the corresponding results.(iv) All the codes developed by the authors.

## Figures and Tables

**Algorithm 1:**
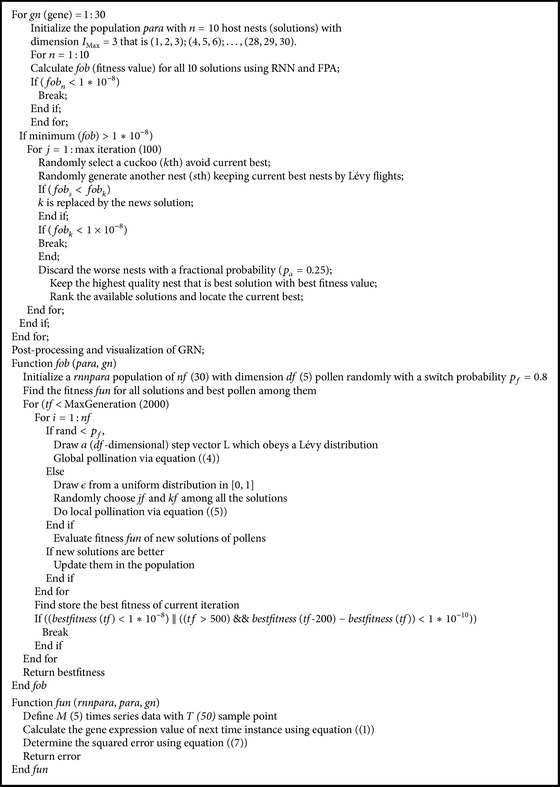


**Table 1:** RNN model parameters of large artificial network [11].

*w*_*i*,*j*_	*w*_1,14_ = −15, *w*_5,1_ = 10, *w*_6,1_ = −20, *w*_7,2_ = 15, *w*_7,3_ = 10, *w*_8,4_ = 20, *w*_9,5_ = −20, *w*_9,6_ = 10, *w*_9,17_ = 10, *w*_10,7_ = −10, *w*_11,4_ = −15, *w*_11,7_ = 15, *w*_11,22_ = −15, *w*_12,23_ = 10, *w*_13,8_ = 20, *w*_14,9_ = 15, *w*_15,10_ = −10, *w*_16,11_ = 15, *w*_16,12_ = −15, *w*_17,13_ = −20, *w*_19,14_ = −15, *w*_20,15_ = 10, *w*_21,16_ = −20, *w*_23,17_ = −10, *w*_24,15_ = −15, *w*_24,18_ = −20, *w*_24,19_ = 15, *w*_25,20_ = −10, *w*_26,11_ = 20, *w*_26,28_ = 20, *w*_27,24_ = −15, *w*_27,25_ = 10, *w*_27,30_ = 15, *w*_28,25_ = −15, *w*_29,26_ = 10, *w*_30,27_ = 15, others *w*_*i*,*j*_ = 0.0

*β*_*i*_	*β*_*i*_ = 5 for *i* = {2,5, 6,10,16,24,28}, *β*_*i*_ = −5 for *i* = {15,17,27} otherwise *β*_*i*_ = 0

*τ*_*i*_	*τ*_*i*_ = 10 for *i* = {1,2,…, 30}

**Table 2:** A comparative study of the performance of the large-scale artificial network.

Data type	Method	TP	TN	FP	FN	*S*_*n*_	*S*_*p*_	Accuracy	MCC
Noiseless	CS-FPA	32	860	4	4	0.889	0.995	0.991	0.884
	DE [11]	22	861	3	14	0.611	0.996	0.981	0.725

5% noise	CS-FPA	32	845	19	4	0.889	0.978	0.974	0.735
	DE [11]	11	848	16	25	0.305	0.981	0.954	0.329

**Table 3:** Results obtained for the *E. coli* SOS DNA repair network.

	*uvrD*	*lexA*	*umuDC*	*recA*	*uvrA*	*uvrY*	*ruvA*	*polB*
*uvrD*	0	**−**	0	0	0	0	0	0
*lexA*	+	0	0	0	0	+	0	0
*umuDC*	**−**	**−**	0	0	0	0	0	0
*recA*	0	**−**	0	0	**−**	0	0	0
*uvrA*	0	0	+	0	0	0	**−**	0
*uvrY*	0	0	0	**−**	0	0	0	+
*ruvA*	0	**−**	0	0	0	0	0	+
*polB*	0	0	**−**	**−**	0	0	0	0
