# Supplementary material for: Large-Scale Recurrent Neural Network Based Modelling of Gene Regulatory Network Using Cuckoo Search-Flower Pollination Algorithm
Source: Adv Bioinformatics. 2016 Feb 16;2016:5283937. doi: 10.1155/2016/5283937 (PMC4771889; doi:10.1155/2016/5283937)
Supplement: Supplementary file 1 — We have included the following in the supplementary materials: (i) The noiseless dataset of the artificial network, used for training, and the corresponding results.(ii) The dataset of the artificial network with 5% noise added, and the corresponding results.(iii) The dataset of the E. coli DNA SOS repair network, and the corresponding results.(iv) All the codes developed by the authors. [file 5283937.f1.zip › Contribution of Authors.docx]

Contribution of authors:

1. Sudip Mandal: He is responsible for proposing CS-FPA hybrid algorithm, Coding for Artificial Data and writing, final revision the manuscript.

2. Abhinanan Khan: He is responsible to deal with E.Coli data and corresponding validation using CS-FPA and to write up the E.Coli section of the manuscript. Total Initial revision work as per reviewer comment has been jointly executed by S. Mandal and A. Khan

3. Dr. G Saha and Dr R. K. Pal are acting as joint supervisor and supervisor respectively for the proposed research investigation on construction of Gene Regulatory Network using computational methodology. All the necessary works, revision are executed under their supervision and advice.
